# microRNAs: An Emerging Paradigm in Lung Cancer Chemoresistance

**DOI:** 10.3389/fmed.2015.00077

**Published:** 2015-11-04

**Authors:** Srivatsava Naidu, Michela Garofalo

**Affiliations:** ^1^Transcriptional Networks in Lung Cancer Group, Cancer Research UK Manchester Institute, University of Manchester, Manchester, UK

**Keywords:** chemoresistance, lung cancer, microRNAs, drug response, cancer therapy

## Abstract

Lung cancer is considered the most deadly of all cancers, with limited therapeutic options. Although advanced drugs have been tried in clinic, the therapeutic success has largely been hampered due to rapid development of drug-resistance mechanisms. Recently, microRNAs (miRNAs), a class of small non-coding RNAs, have occupied center stage in cancer biology. miRNAs negatively regulate gene expression either by promoting degradation or by interfering with translation of messenger RNA targets. Several lines of evidence have confirmed the crucial role of miRNAs in carcinogenesis, and, importantly, in the acquisition of resistance to chemotherapeutics. Modulation of miRNA expression levels has been proven to increase the efficacy of genotoxic drugs in various preclinical cancer studies. Therefore, comprehensive understanding of the role(s) of these key players in drug resistance may provide novel opportunities to design effective combinatorial therapeutic strategies for cancer treatment. In this review, we highlight recent findings on miRNAs acting as oncomiRs and tumor suppressor genes in lung cancer. Moreover, we discuss the involvement of miRNAs in different mechanisms of drug resistance in this deadly disease.

## Introduction

Lung cancer (LC) dominates cancer incidence and related mortality rates globally (1, 2). Histo-logically, LC is classified into two major types: small cell lung cancer (SCLC) and non-small cell lung cancer (NSCLC) (3). SCLC is the most aggressive subtype accounting for 15% of cases, whereas NSCLC, which accounts for 85% of cases, includes adenocarcinoma, squamous cell carcinoma, large cell carcinoma, and other rare subtypes (4). NSCLC and SCLC are generally characterized as different diseases owing to their distinct histological and pathological phenotypes. However, based on case studies and clinical observations, the transformation of NSCLC to SCLC has recently been proposed. Intriguingly, this transformation has partly been attributed to the epidermal growth factor receptor (EGFR) inhibition [for mechanism see Ref. (5)]. Surgical resection is effective for early-stage non-metastatic lung tumors (6). However, chemotherapy, alone or in combination with radiation, is considered as the frontline strategy for the treatment of advanced or metastatic stages of LC (7). Extensive molecular profiling studies have identified several druggable targets for LC therapy. A range of highly effective therapeutic molecules specifically targeting oncogenic mutations and/or signaling pathways driving lung carcinogenesis have been developed and successfully tested in the clinical setting (8). In particular, receptor tyrosine kinase inhibitors (TKIs), which interfere with growth factor receptor signaling in cancer, have shown excellent therapeutic outcome with remarkable clinical benefits (9). However, due to inherent or acquired drug resistance, the efficacy of chemotherapy has been ephemeral and drastically limited, resulting in poor survival rate (10, 11). Various molecular mechanisms contribute to drug resistance, including alterations in drug targets, elevated drug efflux, mutations restoring DNA repair function, activation of alternative survival signaling cascades, and deregulated apoptosis (12). Although the molecular events of drug resistance have been significantly investigated, the broad spectrum of resistance mechanisms remains largely enigmatic. Several lines of evidence have strongly correlated aberrant miRNA expression to the etiology of various cancers (13), including LC (14). Importantly, selective modulation of miRNA activity can improve the response to chemotherapy (15). This review summarizes the recent advances on the role of miRNAs in regulating mechanisms of drug resistance and also debates their potential as a therapeutic option to evade chemoresistance in LC.

## microRNA: Biogenesis and Function

microRNAs (miRNAs) are endogenously expressed small non-coding RNAs that are highly conserved in eukaryotes (16). The canonical miRNA biogenesis largely mimics classical protein-coding gene transcription mechanism. RNA polymerase II transcribes miRNA genes to primary miRNAs, which are processed to pre-miRNAs in the nucleus by an RNase III endonuclease–Drosha microprocessor complex (17). Pre-miRNAs are exported to the cytoplasm by Exportin-5–Ran-GTP complex (18) and are subsequently cleaved by Dicer1 to form a mature miRNA duplex (19). The guide strand in the duplex, along with Argonaute protein AGO2, is configured into a miRNA-induced silencing complex (miRISC) and the passenger strand is degraded (20).

The miRISC binds to the complementary sequence within the 3′ untranslated regions (UTRs) of target messenger RNAs (mRNAs). Depending on the percentage of complementarity, the sequence-specific binding of miRISC influences the degradation or the level of translation of target mRNAs (21). Research so far has confirmed the involvement of miRNAs in the regulation of fundamental cellular processes such as cell growth and differentiation, cell cycle control, proliferation, apoptosis, and tissue development (22, 23). Intriguingly, a single miRNA can regulate multiple mRNA targets; conversely, certain mRNAs can cooperatively be targeted by several miRNAs, underscoring the complexity involved in miRNA-mediated gene regulation (24). Importantly, genetic manipulations affecting miRNA biogenesis, as a consequence of reduced total miRNA output, have been shown to cause oncogenic phenotype in various experimental models (25). Therefore, it is highly plausible that aberrant miRNA expression can alter normal cellular behavior, leading to carcinogenesis.

## microRNAs in Lung Cancer

Kumar et al. have demonstrated that conditional knockout of Dicer1 enhanced tumor development in a K-Ras-driven LC mouse model (26). Also, reduced Dicer expression has been linked to poor survival of NSCLC patients (27). Together, these studies highlight the importance of miRNA expression in maintaining homeostasis in lung tissues. In a recent study, differential miRNA expression profiles have been reported for SCLC and NSCLC cells compared to their normal counterparts. This study indicates a progressive trend in dysregulation of miRNA expression from normal to NSCLC cells to SCLC cells, suggesting that increased miRNA dysregulation may play a role in progression toward more malignant phenotypes (28). Also, specific miRNA signatures have been correlated with disease-free survival in NSCLC patients (29). Both oncomiRs and tumor suppressor miRNAs have been reported. We briefly describe well-characterized miRNAs from each category in Table 1.

**Table 1:** **microRNAs involved in lung cancer**.

Function	miRNA	Targets
**OncomiRs**		
SCLC	miR-25	Cyclin E2 and CDK2
NSCLC	miR-21	Apaf1, Faslg, RhoB, TPM1, PDCD4, PTEN
	miR-17/92	HIF-1a, PTEN, BCL2L11, CDKNA
	miR-31	TSP-1
	miR-224	PPP2R2A, LATS2, SMAD4
**Tumor suppressor miRs**		
SCLC	miR-34	–
	miR-138	H2AX
	miR-126	SLC7A5
NSCLC	Let-7	K-RAS, MYC, HMGA2, CDK6, cyclinD2, CDC25A
	miR-34b	MET, MYC, BCL2

*microRNAs acting as oncogenes or tumor suppressor genes in lung cancer (SCLC and NCSLC) and their respective targets are reported*.

### OncomiRs in Lung Cancer

miR-21 is frequently upregulated in LC and has been correlated with poor prognosis of NSCLC patients (30). The majority of validated targets for miR-21 are tumor suppressors, including the proapoptotic Apaf1, Faslg, and RhoB. The deletion of miR-21 in a K-rasLA2 mice (harboring latent K-ras G12D allele activated by two recombination events) model significantly reduced tumor burden, thus confirming its oncogenic role in LC (31). Also, miR-21 downregulates the tumor suppressor PTEN, enhancing tumor growth and invasion in NSCLC (32). The expression of miR17/92a cluster (comprising miR-17-3p, miR-17-5p, miR-18a, miR-19a, miR-20a, miR-19b-1, and miR-92a) has also been shown to be higher in LC. This oncogenic cluster primarily targets HIF-1a, PTEN, BCL2L11, CDKNA, and TSP-1, causing neovascularization and proliferation. Specific inhibition of miR-17-5p and miR-20a markedly induced apoptosis in A549 cells (33). miR-31 exerts oncogenic effects by targeting PPP2R2A and LATS2, causing activation of alternative growth pathways in LC (34). More recently, Cui et al. have demonstrated the oncogenic role of miR-224 in NSCLC. In this study, the authors show that miR-224 targets TNFα-induced protein 1 and SMAD4, thereby promoting proliferation, migration, and invasion both *in vivo* and *in vitro* (35). miR-25 is shown to be overexpressed in SCLC cells and SCLC tumor samples. Down modulation of miR-25 significantly reduced cancer cell growth and invasion capacities of SCLC cells lines (36).

### Tumor Suppressor miRNAs

Lethal-7 (let-7) is the first miRNA to be linked with LC (37), and reduced expression of let-7 has been correlated with poor survival rate of LC patients (38). Importantly, let-7 suppresses the expression of key oncogenes, such as K-RAS (39), MYC (40), and HMGA2 (41), suggesting a crucial tumor suppressor role for this miRNA. As expected, ectopic expression of let-7 significantly reduced tumor burden in various *in vitro* and *in vivo* studies (42, 43). The expression of miR-34 has also been reported to be lower in LC (44). miR-34 targets prototypical oncogenes such as MET, MYC, and BCL2, thus acting as tumor suppressor (45). Furthermore, Kasinski and Slack have shown that enforced expression of miR-34 dampened tumor growth in a K-ras:p53 (Kras^LSL-G12D/+^;Trp53^LSL-R172H/+^) mouse model (46). The same laboratory demonstrated that nanoparticle-mediated delivery of miR-34 and let-7 significantly reduced tumor growth and prolonged the survival of a K-ras:p53 NSCLC mice model (47). Independently, miR-34 family expression has shown to be reduced by methylation in SCLC cell lines (H1048 and SBC5), and this repression was rescued after 5-aza-2′-deoxycytidine treatment. Forced expression of miR-34b/c in H1048 and SBC5 cell lines dampened cell growth, migration, and invasion compared with controls (48). Together, these studies strongly suggest the potential therapeutic advantage of let-7 and miR-34 in LC. Also, miR-138 (by targeting H2AX expression) (49) and miR126 (by targeting SLC7A5) (50) remarkably reduced growth and proliferation in SCLC cell lines. Similarly, miR-200 family (regulating metastasis) (51) and miRNA-29 family (involved in epigenetic regulation of gene expression) (52) have been reported as tumor suppressor miRNAs in LC.

## Role of miRNAs in Lung Cancer Chemoresistance

**Figure 1:**
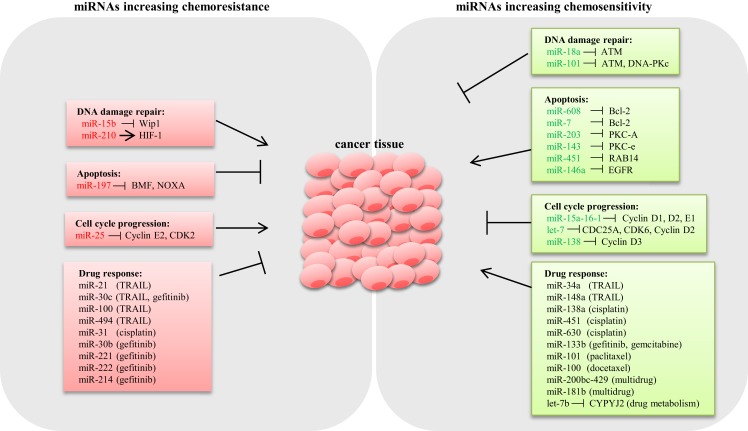
**Schematic representation of miRNAs involved in lung cancer chemoresistance**. Several miRNAs have shown to modulate the expression of key genes involved in chemoresistance mechanisms in lung cancer. miRNAs conferring chemoresistance are shown in red, and miRNAs responsible for enhancing drug response are shown in green.

Drug resistance is considered as a primary cause for chemotherapeutic failure (53). miRNA dysregulation affects the expression of genes involved in drug-resistance mechanisms such as DNA damage repair, apoptosis, and cell cycle control (Figure 1).

### DNA Damage Repair

DNA damage repair (DDR) is an intrinsic cellular mechanism triggered in response to genomic injury caused by factors such as ionizing radiation (IR), UV, and genotoxic drugs. Cells respond to DNA damage by halting cell cycle progression, and depending on the damage type, various repair mechanisms are activated (54). However, if the damage is beyond repair, cells undergo apoptosis (55). Inadequate DDR capacity is considered as a common trait for cancer cells. Several studies have demonstrated that various miRNAs modulate the expression of DDR pathway components in LC. Shin et al. analyzed the expression profile of IR-responsive miRNAs in A549 lung carcinoma cells and revealed a list of miRNAs that are differentially expressed. Further qPCR analysis confirmed that miR-16-2, miR-106a, miR-139-3p, and miR-516a-5 are significantly downregulated in response to IR. Target prediction for these IR-responsive miRNAs suggested that the majority of the potential targets are involved in DDR, cell cycle regulation, and apoptosis (56). Rahman et al. demonstrated that miR-15b expression is induced by IR, causing G2/M arrest and increased DDR response in human bronchial epithelial cells. In this study, overexpression of miR-15b resulted in activation of ATM/ATR pathway and enhanced DDR response, suggesting a causal role of miR-15b for radioresistance (57). It is well known that hypoxia induces resistance to both chemotherapy and radiotherapy (58). A study by Huang et al. showed that HIF-1 transcriptionally activates miR-210 in hypoxia conditions (59). Grosso et al. demonstrated that LC cells under hypoxic conditions exhibited reduced apoptosis in response to radiation compared to cells cultured in normoxia as a consequence of miR-210 upregulation. Abrogation of HIF-1 in cells stably expressing miR-210 rescued the resistant phenotype, confirming that the mechanism of resistance is dependent on HIF-1. Therefore, miR-210 could potentially be an ideal candidate to enhance radiosensitivity in lung tumors (60). By contrast, miR-18a expression is downregulated in IR-resistant LC cells (61). Overexpression of miR-18a reduced ATM expression, thus enhancing radiosensitivity in NSCLC cells (62). Also, miR-101 has shown to sensitize LC cells to radiation by targeting the expression of ATM and DNA-PKc expression (63) (Figure 1).

### Apoptosis

Aberrant apoptosis is considered as a major contributing factor for tumor progression and chemoresistance (64). Research so far suggested that dysregulated miRNAs modulate the expression of genes related to apoptosis, thus playing an important role in drug resistance (65). The inverse relation between the antiapoptotic gene Bcl-2 and miR-608 has been reported in LC cell lines. Enforced expression of miR-608 markedly increased apoptosis, suggesting a proapoptotic role of miR-608 in LC (66). Also, miR-7 has been shown to target Bcl-2, resulting in a significant increase in caspase-3/7 activity in A549 cells (67). miRNAs modulating prosurvival signaling pathways such as PKC, AKT, and ERK1/2 have been reported in LC. For example, miR-203 and miR-143 target PKC-A and PKC-e, thus enhancing apoptosis (68, 69). miR-451 induces apoptosis by targeting RAB14. Downregulation of RAB14 by miR-451 reduced Akt phosphorylation, which led to the accumulation of the proapoptotic Bax protein (70). Downregulation of EGFR by miR-146a reduced ERK-1/2 activity in A549 cells (71), whereas knockdown of miR-197 restored proapoptotic BMF and NOXA expression and induced apoptosis (72) (Figure 1).

### Cell Cycle Control

microRNAs modulate cell proliferation by targeting key components associated with cell cycle. For instance, the miR-15a-16-1cluster has been shown to silence cell cycle regulators such as cyclin D1, D2, and E1, thus inducing G1–G0 arrest in NSCLC cells (73). Let-7, a tumor suppressor frequently deleted or downregulated in LC, has been shown to negatively regulate the expression of cell cycle progression genes such as CDC25A, CDK6, and cyclin D2 (74). Also, overexpression of miR-138 silenced cyclin D3, leading to cell cycle arrest in A549 cells (75). Conversely, downregulation of miR-25 induced cell cycle arrest in the G1 phase through the downregulation of cyclin E2 and CDK2 in SCLC cells. Interestingly, reconstitution of cyclin E2 reversed the cell cycle arrest phenotype in H510A cells, suggesting that the oncogenic role of miR-25 is cyclin E2 dependent (36) (Figure 1).

### Modulation of microRNAs and Response to Chemotherapy

Altered expression of miRNAs modulates the expression of drug target proteins and/or activates alternative compensatory pathways, leading to drug resistance in cancer. Several studies have reported the role of miRNAs in modulating the response to different drugs.

#### TNF-Related Apoptosis-Inducing Ligand

TNF-related apoptosis-inducing ligand (TRAIL) is a cytokine belonging to the TNF superfamily, which induces apoptosis specifically in cancer cells sparing the normal cells. In clinical trials, TRAIL has shown to be effective only in a very small subset of LC patients (76), but, unfortunately, the majority of lung tumors are TRAIL resistant and the causes of this resistance are mostly unknown. We showed that miR-34a/c inhibited the expression of oncogenic PDGFR-α and PDGFR-β, and overexpression of miR-34a/c enhanced TRAIL sensitivity in LC cell lines (77). In another study, we also demonstrated that miR-148a sensitized cells to TRAIL and reduced lung tumorigenesis (both *in vitro* and *in vivo*) through the downregulation of matrix metalloproteinase 15 (MMP15) and Rho-associated kinase 1 (ROCK1) (78). Acquired TRAIL resistance has been linked to miR-21, miR-30c, and miR-100 expression in NSCLC. Indeed, continuous exposure to subtoxic concentrations of TRAIL induced acquired resistance to the drug and activation of NF-kB p65, which in turn transcriptionally activates miR-21, miR-30c, and miR-100. These three miRNAs are responsible for the resistant phenotype by silencing important tumor suppressor genes such as caspase 8, caspase 3, Foxo3A, and TRAF-7 (79). Also, ERK1/2-dependent upregulation of miR-494 has shown to induce TRAIL resistance in NSCLC by targeting BIM expression (80).

#### Cisplatin

Cisplatin binds to and causes cross-linking of DNA, which ultimately triggers apoptosis and is generally considered as first-line therapy for NSCLC. The modulatory role of miRNAs involved in cisplatin response in LC has been demonstrated in various studies. Wang et al. analyzed the expression profile of miRNAs in a cell line with acquired cisplatin resistance (A549/DDP) and demonstrated that upregulation of miR-138 increased sensitivity to the drug and enhanced apoptosis. The authors also demonstrated that excision repair cross-complementation group 1 (ERCC1) is a target of miR-138, suggesting a crucial role of this miRNA in the acquirement of cisplatin resistance in NSCLC (81). Forced expression of miR-451 has shown to improve cisplatin sensitivity by inhibiting cell growth and inducing caspase-3-dependent apoptosis in A549 cells (82). Also, overexpression of miR-630 upregulated cyclin-dependent kinase inhibitor 1B or p27(Kip1), resulting in G0–G1 phase arrest and significant reduction in proliferation of A549 cells (83). Conversely, oncogenic miR-31 has been demonstrated to induce cisplatin resistance in NSCLC cell lines. Transfection of miR-31 mimics into cisplatin-sensitive SPC-A-1 cells markedly increased resistance to cisplatin. This resistance-causing phenotype has been attributed to the downregulation of the membrane transporter ABCB9 (84).

Recently, expression profiles of miRNA in sensitive and multidrug-resistant SCLC cell lines have been analyzed, and the differential expression of miR-134 has been correlated to drug resistance. miR-134 negatively regulates multidrug-resistance protein MRP1/ABCC1 expression, and forced expression of miR-134 markedly enhanced sensitivity to cisplatin, etoposide, and doxorubicin in H69AR (multidrug-resistant SCLC) cells (85). However, in more aggressive SCLC, the data regarding miRNA dysregulation and corresponding effects on drug resistance are relatively limited compared to extensively studied NSCLC. Comparative analysis of miRNAs dysregulation in all subtypes of LC would greatly help in comprehensive understanding of the role of miRNAs in LC.

#### Tyrosine Kinase Inhibitors

Tyrosine kinase inhibitors, such as gefitinib and erlotinib, block the EGFR and have been shown to be very effective in LC patients with EGFR activating mutations such as deletion in exon 19 or point mutations in exon 21. However, even the patients who respond well to the therapy in the beginning become resistant later. The causes of this resistance are another mutation in the tyrosine kinase domain of the EGFR (T790M mutation) or MET amplification. The modulatory role of miRNAs in the EGFR signaling pathway of lung carcinogenesis and target therapy is gaining importance. We demonstrated that miR-30b, miR-30c, miR-221, and miR-222 are regulated by EGF and MET receptors. Upregulation of these miRNAs induced gefitinib resistance in LC cells by the modulation of BIM, PTEN, and APAF-1 expression. Interestingly, MET inhibition decreased the expression of these miRNAs, thus conferring increased sensitivity to the drug (86). Oncogenic miR-214 appears to be a contributing factor for gefitinib resistance in adenocarcinoma cells. The inhibition of miR-214 caused an upregulation of PTEN expression and inactivation of AKT (87). On the other hand, miR-133b directly targets EGFR, and overexpression of miR-133b in PC-9 and A549 cells inhibited phosphorylation of EGFR, AKT, and extracellular signal-related kinase (ERK)1/2. Importantly, miR-133b was able to restore EGFR-TKI sensitivity in TKI-resistant NSCLC cells (88).

### Taxanes

The involvement of miRNAs in taxanes (mitotic inhibitors)-related resistance in LC has been reported in various studies. Zhang et al. have identified an inverse relation between miR-101 and the oncogene EZH2 in NSCLC. Overexpression of miR-101 downregulated EZH2 expression and sensitized NSCLC cells to paclitaxel (89). miR-100 has been shown to reduce the expression of PLK1 and increase chemosensitivity to docetaxel (90). Also, miR-133b modulates the response to gemcitabine (nucleoside antimetabolite) in NSCLC cells. miR-133b targets antiapoptotic proteins such as Bcl-w and Mcl-1. Importantly, a combinatorial treatment of miR-133b with gemcitabine significantly enhanced apoptosis in NSCLC cells (91). Transfection of miR-200bc-429 (92) and miR-181b (93) into A549 cells significantly increased apoptosis compared to parent A549 cell line through the targeting of Bcl2 and XIAP. Recently, Chen and colleagues have demonstrated that the expression of drug metabolizing enzyme cytochrome P450 epoxygenase 2J2 (CYP2J2) is inversely proportional to the tumor suppressor let-7b expression in squamous cell lung cancer. Vector-mediated overexpression of let-7b in tumor-engrafted mice led to the downregulation of CYP2J2 and significant reduction in tumor growth (94).

## Future Perspectives

Drug resistance compromises the therapeutic benefits of chemotherapy and remains a major challenge to overcome in LC treatment. Therefore, research efforts should aim to find novel strategies toward the ultimate solution for drug resistance. During the last two decades, a continuous stream of reports unanimously confirmed the crucial role of miRNAs in carcinogenesis and miRNAs emerged as key players in modulating sensitivity and resistance to chemotherapy. Modulating the expression of miRNAs has shown to increase drug sensitivity in various *in vitro* and *in vivo* studies, suggesting that a combinatorial therapeutic option (miRNAs and chemotherapy) could be useful for resistant phenotypes. Nevertheless, it is important to consider that miRNA expression patterns and corresponding effects are tissue and cell type specific. Given the heterogenous nature of tumors, it would be necessarily essential to take into account of the miRNA expression profiles of various cell types constituting normal and tumor tissues. Probing miRNA-dependent phenotypes in inappropriate cell types may lead to erroneous conclusions. For instance, studies performed in epithelial cell lines derived from colon cancer suggested miR-143/145 as tumor suppressor in colon cancer. However, new data indicate that miR-143/145 is primarily mesenchymal, with no suggestive functions in intestinal epithelial cells (95). These discrepancies underpin the importance of “context” while investigating and interpreting the role of miRNAs in cancer. Specific miRNA signatures associated with drug resistance can be used as biomarkers for disease stratification, helping in the design of new strategies for personalized treatments. However, the use of miRNAs as therapeutic tools is still in its infancy. One of the major obstacles for miRNA therapy is the efficient and specific delivery to the tumor sites. Technological advancements on miRNA delivery front look promising. Therefore, exploiting miRNAs for cancer therapy, either alone or in combination with conventional chemotherapy regimens, may have more efficient clinical outcome particularly for chemoresistant lung tumors.

## Author Contributions

SN drafted the manuscript and designed the figures. MG conceived the study, guided in its design, and edited the text. All authors read and approved the final manuscript.

## Conflict of Interest Statement

The authors declare that the research was conducted in the absence of any commercial or financial relationships that could be construed as a potential conflict of interest.
